# Supplementary material for: Synthesis of Poly(Trimethylene Carbonate) from Amine Group Initiation: Role of Urethane Bonds in the Crystallinity
Source: Polymers (Basel). 2021 Jan 16;13(2):280. doi: 10.3390/polym13020280 (PMC7829917; doi:10.3390/polym13020280)
Supplement: Supplementary file 1 [file polymers-13-00280-s001.pdf]

Supporting Materials

Typical procedure for polymerization of TMC initiated by ROP-amine with MSA in one step.
Propylamine (1 eq.) and MSA (0.2 eq.) were added into a solution of TMC (100 eq.) in CH2Cl2 (1.0 mol.l-1). Then the mixture was stirred at room temperature until the complete consumption of the amine. After 24 hours then reaction was uncompleted (conv. 50%), the crude solution was precipitated in methanol.
^{1}H NMR (δ in ppm, $CDCl_3$, 400 MHz): 4.4 (t, CH_2 TMC), 4.2 (t, CH_2), 4.0 (m, CH_2), 3.8 (m, CH_2), 3.0 (MSA), 2.2 (q, CH_2 TMC), 2.0 (q, CH_2), 1.8 (m, CH_2), 1.0 (t, CH_3).

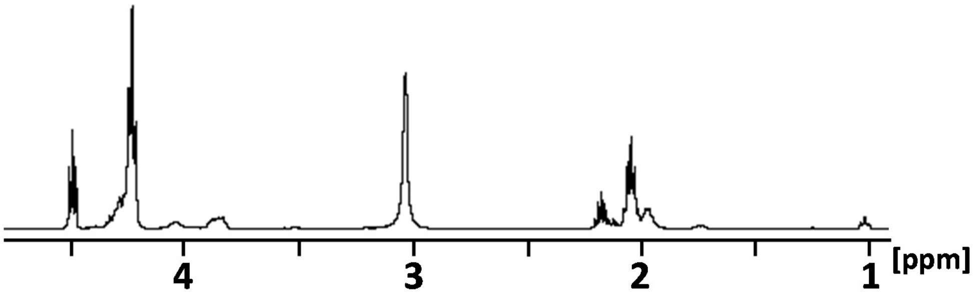

Figure S1. ^{1}H NMR at 24 hours in one step of PTMC with MSA ($CDCl_3$).

Typical procedure for polymerization of TMC initiated by ROP-amine with TBD in one step
1,6-Hexanediamine (1 eq.) and TBD (0.2 eq.) were added into a solution of TMC (100 eq.) in CH_2Cl_2 (1.0 mol.l^{-1}). Then the mixture was stirred at room temperature until the complete consumption of the amine monitoring by ^{1}H NMR. After complete conversion, TBD was quenched with benzoic acid. The crude solution was precipitated in methanol.
^{1}H NMR (δ in ppm, $CDCl_3$, 400 MHz): 4.2 (t, CH_2), 3.7 (m, CH_2), 2.0 (q, CH_2).

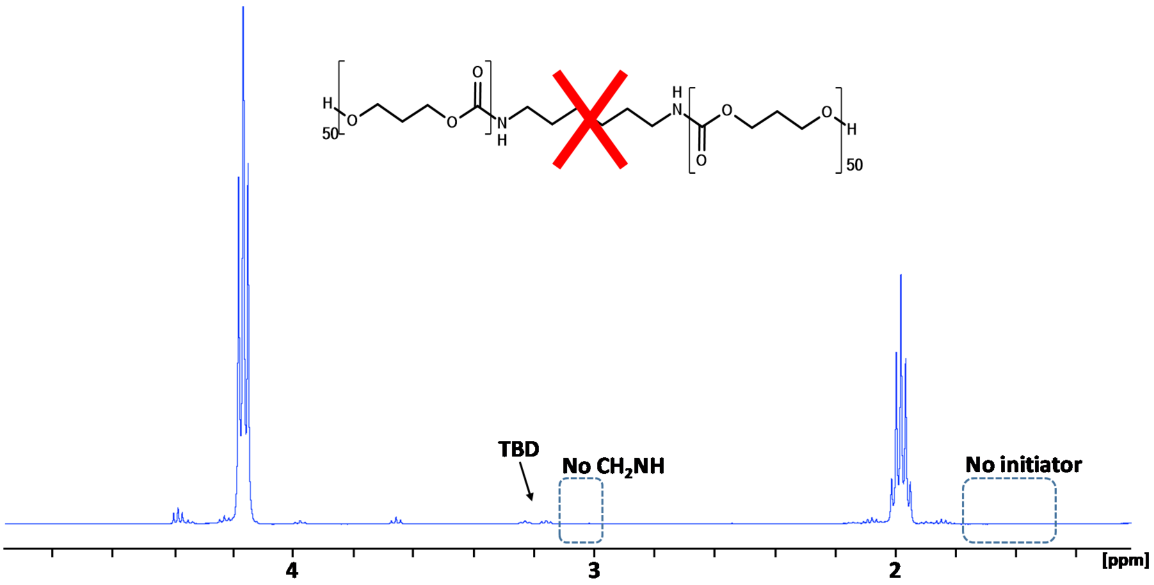

Figure S2. ^{1}H NMR in one step of PTMC initiated by 1,6-hexanediamine with TBD ($CDCl_3$).

Typical procedure for polymerization of TMC initiated by ROP-amine with TBD in one pot two step

Propylamine (1 eq.) was added into a solution of TMC (100 eq.) in CH_2Cl_2 (1.0 mol.L^{-1}). The mixture was stirred at 30 °C until the complete consumption of the amine. Then a solution of TBD (0.2 eq.) in CH_2Cl_2 was added to the reaction mixture. After complete reaction, TBD was quenched with benzoic acid. The crude solution was precipitated in methanol.
^1H NMR (δ in ppm, $CDCl_3$, 400 MHz): 4.7 (m, 1 H, NH), 4.2 (t, 40 or 400 H, CH_2), 3.8 (m, 2 H, CH_2), 3.4 (TBD), 3.2 (m, 2 H, CH_2), 2.0 (q, 20 or 200 H, CH_2), 1.5 (m, 2 H, CH_2), 0.9 (t, 3 H, CH_3).

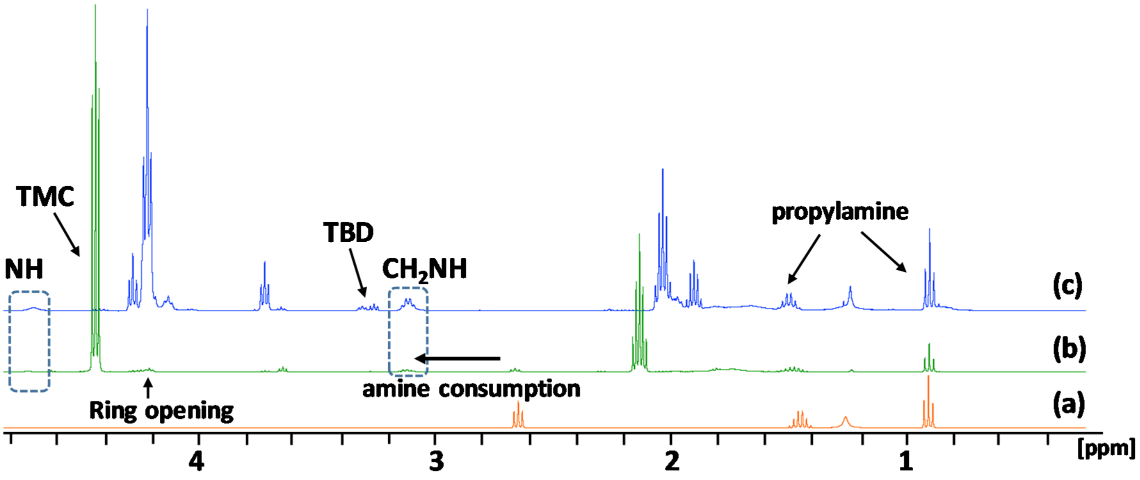

Figure S3. ^1H NMR in one pot two steps of (a) propylamine, (b) crude after 6 hours at 30 °C and (c) PTMC after addition of TBD ($CDCl_3$)

Typical procedure for functionalization of PTMC end chain by urethane

The urethanisation of the PTMC terminal groups achieved with propyl isocyanate (Sigma-Aldrich, France). PTMC (1 eq.) was dissolved in CH_2Cl_2 (1.0 mol.L^{-1}) before adding propyl isocyanate (3 eq.). The reaction was catalyzed with 0.8 wt.% of dibutyltin dilaurate (DBTDL) from Sigma-Aldrich (Saint-Quentin-Fallavier, France). The solution was carried out at 60 °C under magnetic stirring for 2 hours. The polymer was recovered by precipitation in methanol.
^1H NMR (δ in ppm, $CDCl_3$, 400 MHz): 4.7 (m, 1 H, NH), 4.2 (t, 40 or 400 H, CH_2), 3.2 (m, 4 H, CH_2), 2.0 (q, 20 or 200 H, CH_2), 1.5 (m, 4 H, CH_2), 0.9 (t, 6 H, CH_3).

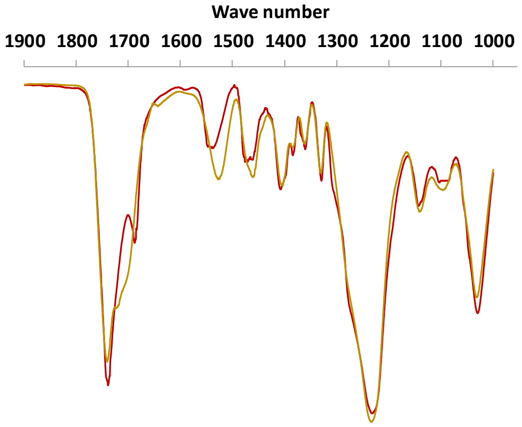

Figure S4. FT-IR spectra in cm^{-1} of $PTMC_{D\text{-}NH}$ (red) and $PTMC_{F\text{-}NH}$ (yellow)

Table S1. Thermal properties of $PTMC_{D\text{-}NH}$ (DP_n=100) after 24 hours, 2 weeks, 4 weeks and 16 weeks.

**Time**	**T_g (°C)**	**T_m (°C)**	**ΔH_m (J.g^{-1})**
24 hours	-31.3	-	-
2 weeks	-30.6	37.0	25.6
4 weeks	-25.0	40.8	52.5
16 weeks	-27.0	38.3	50.6

Table S2. Thermal properties of $PTMC_{F\text{-}NH}$ (DP_n=100) after 2 weeks.

**PTMC**	**T_g (°C)**	**T_m (°C)**	**ΔH_m (J.g^{-1})**
$PTMC_{D\text{-}NH}$	-30.6	37	25.6
$PTMC_{F\text{-}NH}$	-31.1	39	46.3

The addition of urethane function by end chain modification with isocyanate increases the number of urethane groups. This leads in IR by a band even more intense at 1523 cm^{-1} and in DSC by an improvement of the crystallization (Figure S4 and Table S2).
